# Supplementary material for: Determinants of age at first sex inequality between women and men youth in Uganda: A decomposition analysis
Source: PLOS Glob Public Health. 2022 Sep 22;2(9):e0000303. doi: 10.1371/journal.pgph.0000303 (PMC10021423; doi:10.1371/journal.pgph.0000303)
Supplement: Attachment — Submitted filename: Response to Reviewers.docx [file pgph.0000303.s001.docx]

**Response to Reviewers**

PGPH-D-22-00175
Determinants of gender inequality in sexual debut among youth in Uganda:  a decomposition analysis
PLOS Global Public Health **Reviewer#1:**  The article needs to be edited and revised by a native speaker of English for readability and coherence. Additionally, there are excess spaces and inappropriate use of punctuation throughout the paper. English usage does not always seem standard.

**Response**	Thank you very much for agreeing to review our manuscript and for the constructive comments. The manuscript was revised.

The chart should be reformatted so that text is not truncated or hyphenated. **Response** Thank you for this observation. We have reformatted the charts.

I appreciated the discussion of limitations, but more attention should be paid on why early marriage of women (and men) was not considered as a potential contributory factor. **Response** Thank you so much for this comment. We did not consider early marriage as a contributory factor because we lacked evidence from the results of the statistical analysis (Oaxaca decomposition analysis). Marital status was not among the factors which explained sexual debut inequality between female and male youth. It was not statistically significant in decomposition analysis.

"Spouse of household" being more often women than men is almost self-evident, so I'm not sure how much of a value that provides as an independent variable. **Response**	Thank you for this observation. The independent variable is “relationship to household head” not	“spouse of household”. Spouse of household is one of the groups in “the relationship to the household head variable”. Following your advice, we have deleted this statement from the manuscript, “From the descriptive results, more female youth were spouses of household heads compared to male youth”, (56.01% female youth vs 0.8% male youth) but we have not deleted the relationship to the household head variable because literature reveals relationship to household head is associated with sexual debut (references are below this statement). Other groups from relationship to household head variable (such as, “daughters/sons of household heads”, other relatives of the household head like cousins, grandchildren, sisters, brothers, etc) which were coded from this variable have policy implications. We could not delete the spouse/wife group from the relationship to the household head independent variable because that was their relationship to the head of the house and being children of the head of the household, being a relative (other relatives e.g., sister, cousin, etc) to the household head was statistically associated with the sexual debut.

Pilgrim, N. A., Ahmed, S., Gray, R. H., Sekasanvu, J., Lutalo, T., Nalugoda, F., Serwadda, D., & Wawer, M. J. (2014). Family structure effects on early sexual debut among adolescent girls in Rakai, Uganda. *Vulnerable children and youth studies*, *9*(3), 193–205. https://doi.org/10.1080/17450128.2013.842027

Davis, E. C., & Friel, L. V. (2001). Adolescent sexuality: Disentangling the effects of family structure and family context. *Journal of marriage and family*, *63*(3), 669-681.

Rizkianti, A., Saptarini, I., & Masitoh, S. (2020). THE EFFECT OF FAMILY LIVING ARRANGEMENT ON DELAYED SEXUAL DEBUT AMONG FEMALE TEENAGERS IN INDONESIA. *Jurnal Kependudukan Indonesia*, *15*(1), 49-58.

Longmore, M. A., Manning, W. D., & Giordano, P. C. (2001). Preadolescent Parenting Strategies and Teens’ Dating and Sexual Initiation: A Longitudinal Analysis. *Journal of Marriage and Family*, *63*(2), 322–335. http://www.jstor.org/stable/3654595

**Finally, setting the outcome of interest as a binary variable (sexual debut younger than, or older than, 18 years) instead of as a continuous variable was an interesting choice. It may have been required by the methodology chosen (****Oaxaca decomposition analysis) but the** **use of a legal rule (18 years) should be justified. Is this age also meaningful statistically and in real-life? Otherwise, the use of a legal rule could be seen as arbitrary if not justified more.**

**Response**

Thank you for the advice. We have added more justification. Using 18 as a cut-off age for sexual debut is also meaningful in real life, it is not only for the legal rule**.** In the Ugandan context, all people below 18 years are regarded as children (1). Older people think that the best protection for children is to abstain from sex until they are18 years (2). Parental consent is also required before they seek health care (3). Using 18 as a cut-off age for sexual debut is good because our results will inform health workers, parents, and policymakers, on the need to adjust some policies to reduce early sexual debut in youth and its health implications like HIV and teen pregnancies. The results can also be used by other countries with similar contexts **(**3**).**

1. Ministry of gender labour and social development, 2020. National child policy. [cited 2022 May 3rd]. Available from: <https://www.unicef.org/uganda/media/8166/file/Final-Uganda-National%20Child%20Policy-October-2020-lores.pdf>.

2.Singh J, Chareka S, 2018. AGE OF CONSENT: LEGAL, ETHICAL, SOCIAL AND CULTURAL REVIEW - UGANDA COUNTRY REPORT.

3. Kyegombe, N., Buller, A.M., Meiksin, R., Wamoyi, J., Muhumuza, R. and Heise, L., 2022. Adolescent girls’ perceived readiness for sex in Central Uganda-liminal transitions and implications for sexual and reproductive health interventions. *Culture, health & sexuality*, *24*(3), pp.391-405.

The paper is compelling, timely, and important to the literature. I would recommend publication of this paper after these concerns are addressed.

**Response**	Thank you very much.

**Reviewer #2:** The paper is taken from what seems to be a dissertation and is not written in an appropriate IMRAD format but matching that of a dissertation. A journal article has a focus that this paper lacks.

**Response**	Thank you so much for this comment. We followed IMRAD format when we were writing this paper. Our paper has introduction, methods, results and discussion and it is not in detail like a dissertation. “IMRAD” format refers to a paper that is structured by four main sections: Introduction, Methods, Results, and Discussion, (Sollaci, L. B., & Pereira, M. G. (2004). The introduction, methods, results, and discussion (IMRAD) structure: a fifty-year survey. *Journal of the Medical Library Association: JMLA*, *92*(3), 364–367).

The tables are labelled ascribing methodological attributes and not representative of the table used. **Response** Thank you for this comment. We have corrected the label of the last table.

The label of table 1 states **weighted descriptive characteristics of the study population and sexual debut.** It shows the characteristics of the study population (not methodological attributes) such as, their education levels, wealth quintiles, age distribution, marital status etc**.** The label of table 3 **is “Table 3. Intermediary analysis:** **Material factors related to early sexual debut.”** The results in this table show factors associated with early sexual debut which is also our title. Examples of factors include secondary education, primary education, white collar jobs, blue collar jobs etc. Including intermediary analysis before the label of the table , **“Material factors related to early sexual debut”,** was to guide the reader of this paper.

Following your advice, we have deleted intermediary analysis which was written at the beginning of the label. We have also corrected the last table.

The use of the word 'gender' needs strengthening through appropriate validation of the variable equivalents in the UDHS data set. **Response** Thank you for this observation. While DHS has other gender variables like domestic violence, female genital cutting, women´s most recent marriage, decisions about children´s health and welfare etc, they were not applicable in our study. We had based our gender variable from the following extract from DHS. Our study focused on the highlighted message from DHS.

“Gender is socially constructed derivative of sex (women and men) and encompasses the different roles, rights, expectations and obligations that society attaches to individuals according to whether they are born with female or male characteristics. **Different rights, expectations translate into differences in relative power and access of resources between women and men.** **Gender makes women and men unequal. Such** **gender differences affect women´s and men´s health because they translate into differences in the kind of work men and women engage in and relative to men, women´s lower control over their bodies and sexuality,** **greater restrictions in accessing material and non-material resources such as knowledge, information and greater constraints in accessing needed health care among other things**”, Extract from DHS.

**Our research question**: 1) Is sexual debut equal between male and female youth? Our study found that sexual debut was not equal between male and female youth, women were disadvantaged. Delayed sexual debut, a health behaviour favoured men. We got the sexual debut variable from DHS dataset.

2) What are the factors explaining the gap in sexual debut between female and male youth? **“…gender differences affect women´s and men´s health because they translate into differences in the kind of work men and women engage in and relative to men, women´s lower control over their bodies and sexuality, greater restrictions in accessing material and non-material resources such as knowledge, information and greater constraints in accessing needed health care among other things**”, DHS extract. Literature guided us in choosing variables from DHS dataset that explain the gender gap in sexual debut e.g., education, employment.

After reading your advice, we have changed our grouping variable from gender to sex (female/woman and male/woman youth). We have also changed our title to, determinants of sexual debut inequality between male and female youth in Uganda:  a decomposition analysis.

Thank you reviewers.
