# Supplementary material for: Determinants of age at first sex inequality between women and men youth in Uganda: A decomposition analysis
Source: PLOS Glob Public Health. 2022 Sep 22;2(9):e0000303. doi: 10.1371/journal.pgph.0000303 (PMC10021423; doi:10.1371/journal.pgph.0000303)
Supplement: Attachment — Submitted filename: renamed_4cc6c.docx [file pgph.0000303.s002.docx]

PGPH-D-22-00175R1
Determinants of sexual debut inequality between male and female youth in Uganda:  a decomposition analysis
PLOS Global Public Health

**Response**	Thank you very much for agreeing to review our manuscript and for the constructive comments. The manuscript was revised.

Reviewer #2: Table 1. Weighted descriptive characteristics of the study population and sexual debut
Table 2: Weighted descriptive characteristics of the study population and sexual debut
Comment: These tables report numbers of male and female youth using decimal places. This is because they have used weights from stata to determine the percentages. However, while reporting this, the practice is to report the actual ‘n’ of cases and using the weighted percentages. The authors need to correct this table by eliminating the decimal places in numbers and also verifying and reporting the actual numbers in the sample appropriately.

**Response.** Thank you for this observation. We have corrected it.

Table 3. Material factors related to early sexual debut
Comment: The authors continue to say that the significant findings are bolded. This is not conventional. The usual way to represent this is “p-values which indicated significance at 0.05 or below have been indicated by using bold font’ or some thing. But not the grammatically incorrect ‘bolded’. This had been mentioned in the earlier review as well.

**Response.** Thank you so much for this comment. We have corrected it.

The authors persist in using the words ‘intermediate analysis’ without labelling it as analysis to identify the variables that have a strong association with age at sexual debut. In so far as they have eliminated in the labelling of tables, this change needs to be reflected in the write up. It continues to be present in the write up – lines 479-480.

**Response.** Thank you so much for this comment. We have revised it.

The authors need to get their paper read by a native English speaker to ensure readability.

**Response.** Thank you for this comment. It was edited.
