# Supplementary material for: Determinants of age at first sex inequality between women and men youth in Uganda: A decomposition analysis
Source: PLOS Glob Public Health. 2022 Sep 22;2(9):e0000303. doi: 10.1371/journal.pgph.0000303 (PMC10021423; doi:10.1371/journal.pgph.0000303)
Supplement: Attachment — Submitted filename: A rebuttal letter to PLOS one.docx [file pgph.0000303.s003.docx]

**A rebuttal letter**

Editor´s points.

**1. Please review your reference list to ensure that it is complete and correct. If you have cited papers that have been retracted, please include the rationale for doing so in the manuscript text or remove these references and replace them with relevant current references. Any changes to the reference list should be mentioned in the rebuttal letter that accompanies your revised manuscript. If you need to cite a retracted article, indicate the article’s retracted status in the References list and include a citation and full reference for the retraction notice.**

Thank you very much editor for this comment. We have reviewed our reference list. We have cross-checked all references; no article has been retracted but some references had wrong links and we have corrected them. Thank you.

**2. Kindly use** **conventional variable labels while describing them in the paper. These can be directly taken from the DHS report for Uganda. Such consistency in labeling the variables are useful when the studies uses data of well know data sets.**

Thank you very much for this comment. We used conventional variables from the Uganda demography health survey of 2016, and we operationalized them on pages 9, 10, and 11 of this manuscript. The only challenge we had was with only 2 conventional variables, “age at first sex” and “women/men” but we defined them and described how we coded/renamed them (page 9). In the DHS, women are 15 to 49 years old, and men are 15-54 years old but, our study population is youth (15-30 years), and this group is not defined in the DHS report. We could not label them as young men and women because the cut-off age of young men and young women is 24 years. Fortunately, the DHS program gives us the mandate to reanalyze its data, to recode it with appropriate names, and make the right interpretation. That is why we had coded, renamed, and grouped these 2 variables; “age at first sex” to early sexual debut/delayed sexual debut (page 9) and “women (15 to 49 years)/men (15-54 years)” to female youth/male youth (15-30 years) (as shown on page 9) in a more clear and understandable form.

Following your advice, we have changed the 2 variables which didn’t have conventional labels to DHS conventional variable labels, and our research topic and aim are below. While describing these 2 variables in the paper, we have also replaced sexual debut with age at first sex, female youth with women youth, and male youth with men youth.

**Research topic:** Determinants of **age at first sex** inequality between **women** and **men** youth in Uganda

**Aim of the study:** To identify factors that explain **age at first sex** inequality between **men and women** Ugandan youth.

But the conventional label “age at first sex inequality between women and men youth” sounds ambiguous to us, it was clearer with “sexual debut inequality between female and female youth”. Fortunately, the DHS program gives researchers the authority to operationalize DHS conventional variable labels, to reanalyze DHS data, recode, rename variables, and make the right interpretations. If it is also unclear to you, we are happy to revise it.

**3. Also, our reviewer is of the opinion that the paper will enormously benefit from copy editing. May I encourage you to do one round of copy editing at your end so that we could proceed further.**

Thank you for this comment. The paper was edited.

**Reviewer #2: The authors have addressed all the comments carefully. However, while labelling the variables, it might be useful to distinguish between the variable -** **age at sexual debut - and the individuals.** **Therefore, while referring to the changes needed in policy, the linkage should be to 'age at sexual debut' and not males.**

Response

Thank you very much for this comment, kindly clarify it. We are not sure whether we have understood your point. According to the aim of our study, “to identify factors that explain sexual debut inequality between male and female Ugandan youth”, we find it hard to write the age of sexual debut only. This study aimed to identify factors that explain sexual debut inequality between male and female Ugandan youth. Results showed sexual debut inequality between female and male youth that favored males. More females were at a disadvantage of having an early sexual debut compared to male youth. About 96.37% of the female-male youth early sexual debut gap was explained by unequal distribution of material, behavior/cultural, psychosocial, and demographic factors between male and female youth. More male youth had the advantage of having material, behavior/cultural, psychosocial, and demographic variables that explained the female-male youth early sexual debut gap compared to female youth. Therefore, while referring to the changes needed in policy, to address early sexual debut and related health outcomes, policymakers must combat inequities in access to material, behavior/cultural, psychosocial, and demographic factors such as education, employment opportunities, access to sexual reproductive information through the internet, and newspapers or magazines between male and female youth. Furthermore, policymakers should encourage household heads and household members to always bond with girls and empower them with sexual reproductive information and monitor them.
